# Association between body size and blood pressure in children from different ethnic origins

**DOI:** 10.1186/1475-2840-11-136

**Published:** 2012-11-05

**Authors:** Marieke LA de Hoog, Manon van Eijsden, Karien Stronks, Reinoud JBJ Gemke, Tanja GM Vrijkotte

**Affiliations:** 1 Department of Public Health, Academic Medical Centre, University of Amsterdam, P.O. Box 22660, 1100 DD, Amsterdam, the Netherlands; 2 Department of Epidemiology, Documentation and Health Promotion, Public Health Service, Amsterdam, the Netherlands; 3 Institute of Health Sciences, VU University, Amsterdam, The Netherlands; 4 Department of Paediatrics, EMGO institute, Institute of Cardiovascular Research, VU University Medical Centre, Amsterdam, the Netherlands

**Keywords:** Blood pressure, Ethnicity, Children, Adiposity, Body size

## Abstract

**Objective:**

To assess associations between body size and blood pressure in children (5-6 years) from different ethnic origins.

**Method:**

Five ethnic groups of the ABCD cohort were examined: Dutch (n=1 923), Turkish (n=99), Moroccan (n=187), Black-African (n=67) and Black-Caribbean (n=121). Data on body-mass-index (BMI), waist-to-height ratio (WHtR), fat-mass-index (FMI), and systolic blood pressure (SBP) and diastolic blood pressure (DBP), were collected. Linear regression analysis with restricted cubic splines was used to examine non-linear associations between body size and blood pressure, adjusted for age, sex, height and birth weight.

**Results:**

Ethnic differences were found in associations of BMI with SBP and DBP (SBP: p=0.001 and DBP: p=0.01) and FMI with SBP (p=0.03). BMI and FMI had a relatively large positive association with SBP in Turkish children (BMI: β=2.46mmHg; 95%CI:1.20-3.72; FMI: β=2.41mmHg; 95%CI:1.09-3.73) compared to Dutch (BMI: β=1.31mmHg; 95%CI:0.71-1.92; FMI: β=0.84mmHg; 95%CI:0.23-1.45). Black-Caribbean and Moroccan children showed high blood pressure with low BMI and FMI. Moroccan children showed higher SBP with high BMI and FMI. WHtR was positively associated with SBP and DBP, similar in all ethnic groups. Generally, strongest associations with blood pressure were found for BMI in all ethnic groups.

**Conclusion:**

Ethnic-specific associations between BMI, and FMI and blood pressure are present at young age, with Turkish children showing the highest increase in blood pressure with increasing body size. The higher blood pressure in the Black-Caribbean and Moroccan children with low BMI needs further research. WHtR or FMI do not seem to be associated more strongly to blood pressure than BMI in any ethnic group.

## Introduction

Elevated blood pressure (BP) during childhood is an important contributor to increased cardiovascular risk in later life, such as atherosclerosis [1]. Therefore, recognition and management of elevated BP should start as early as possible. Factors known to influence BP in children include age, sex and body size [2,3]; more specifically, unfavorable BP patterns are more often found in obese children[4] and in certain ethnic groups [5-9].

The relations between measures of general and central adiposity, e.g. body mass index (BMI), waist circumference (WC), and individual cardiovascular measures, like BP, are often studied in both adults and children [10-14]. These body size measures have been implicated to estimate the risks of diabetes and cardiovascular disease risk factors, such as hypertension and high cholesterol [3,11,14-18].

There are indications that ethnicity may modify the relationship between measures of body size and BP [3,19,20], although this is not always the case [7,21]. Ethnic differences in these associations might be due to differences in body composition, a parameter frequently compared between ethnic groups [22-24]. For example, compared with Caucasians with comparable BMI, people of African descent have less visceral fat [22,23] and South Asians have a higher fat mass index (FMI) with a lower BMI [23].

It is unclear which measure of body size best determines BP during childhood. In children, BMI (as an indicator of overall adiposity) is widely used as a measure to define overweight [25], although the potential value to predict BP remains debatable [12-14]. Other measures, like WC and waist-to-height ratio (WHtR), as indicators of abdominal fat mass, may be more directly correlated with BP in children [14,18]. However, a recent study in England (5,235 children aged 9-12 years) found no evidence that FMI or WC is more strongly associated with BP compared to BMI [13].

Knowledge on the association between body size measures and BP in different ethnic groups of children is limited, especially among populations living in Europe. In daily child healthcare professionals measure weight and length to calculate the child’s BMI to indicate health risk. When WHtR or FMI are better determinants of BP in children (or in specific ethnic groups) it might be useful to use these body size measures for better predicting risk for high BP. Therefore, this study uses a multi-ethnic sample of healthy school-aged children to explore ethnic-specific associations between body size measurements and BP, and examines which body size measure is the strongest determinant of BP.

## Methods

### Subjects

**Figure 1:**
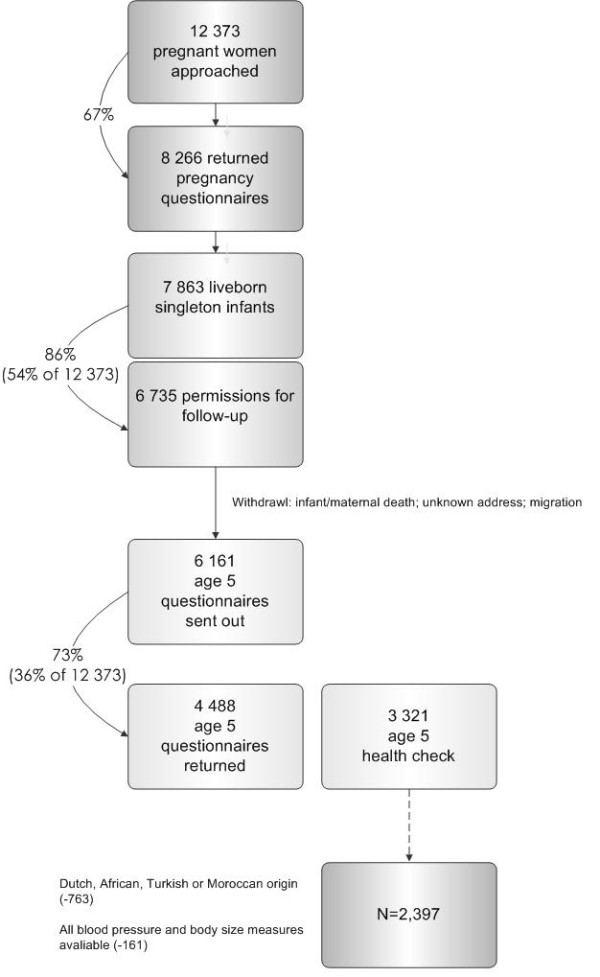
Procedure of the ABCD Study cohort and inclusion in the current analyses.

Data were derived from the Amsterdam Born Child and their Development (ABCD) study. The design and rationale of the ABCD study have been described previously [26]. In brief, during 2003–2004, 12,373 Amsterdam women who first attended antenatal care in Amsterdam were approached to participate in the ABCD study. Of these women, 8,266 (67%) returned the pregnancy questionnaire, which covered sociodemographic characteristics, obstetric history, lifestyles and emotional problems (including multiple psychosocial stress instruments) (phase 1). Of these respondents, 7,863 (95%) women gave birth to a viable singleton infant and 6,735 (86% of 7,863) women gave permission to follow-up. In the following years, the ABCD study covered a questionnaire around three months after birth and the follow-up of growth from the Youth Health Care Centers (phase 2). Phase 3 of the study started in the summer of 2008. Around two weeks after their child’s fifth birthday, 6,161 (91%) mothers who initially gave permission for follow-up with a traceable address, were sent a questionnaire (Dutch, English or Turkish) in which they were also asked for permission regarding participation of their child in the ABCD health check. The questionnaire was returned by 4,488 (73%) mothers who provided information about the child’s health, development and behaviour. Various physical measurements (e.g. height, bodyweight, FMI and BP) took place during a health check (lasting ± 1 h per child) in 3,321 (54%) children. The present study included 2,397 singleton children (aged 5-6 years) from Dutch, Turkish, Moroccan, Black-African or Black-Caribbean origin, for whom the 5-year questionnaire was completed and data about height, bodyweight, WC, FMI and BP were present. The selection of the population included in the current study's analyses (n = 2,397) is visualized in Figure 1.

Ethnicity of the child was based on the country of birth of the child’s mother and her mother, in order to include children from both first-generation (born outside the Netherlands) and second-generation (born in the Netherlands, but with a mother born in another country) mothers. Because they have a similar ethnic background, we combined children from Ghana and other Sub-Saharan African countries in the ‘Black-African’ group, and children from Surinam (Surinam-Creole) and the Antilles in the ‘Black-Caribbean’ group. The groups were composed as follows: Dutch (n= 1,923), Turkish (n=99), Moroccan (n=187), Black-African (n=67) and Black-Caribbean (n=121). Children from ethnic groups other than those mentioned above (n=763) were excluded, because their numbers would be too small to analyse separately. Children with missing data (n=161; mostly missing data on BP) were also excluded.

The study was approved by the review board of all Amsterdam hospitals and the Registration Committee of Amsterdam. All participating mothers gave written consent.

### Measurements

BP was measured with the automatic oscillometric method, using the validated Omron 705 IT (Omron Healthcare Inc, Bannockburn, IL, USA) with a small cuff (arm circumference 17-22 cm) on the non-dominant arm. For BP, first a test measure was made (to comfort/relax) the child followed by a 10-min rest period. Then, BP was measured twice on the right arm in sitting position, with the arm supported at heart level. SBP and DBP (mmHg) were calculated by taking the mean value of the two measures.

For the present study the body size measures were: BMI (kg/m^2^), WHtR and FMI (kg/m^2^). Height was measured to the nearest mm using a Leicester portable height measure (Seca), and weight to the nearest 100 g using a Marsden weighing scale (model MS-4102) to calculate BMI. WC was measured midway between the costal border and the iliac crest to the nearest mm using a Seca measuring tape to calculate WHtR (WC/height). Fat mass was measured with arm-to-leg bioelectrical impedance analysis (BIA) using the Bodystat 1500 MDD (Bodystat Inc. Douglas, UK) [27]. Fat free mass (FFM) was calculated using the total body water (TBW) equation by de Beer [28] (TBW = (0.439*resistancy index) + (0.027*weight) + 4.014) and the tissue hydration constant of Wells et al. [29]. Subsequently, FMI was calculated as (weight -FFM)/height^2^. BMI, WHtR and FMI were standardized to obtain z-scores to enable comparison of anthropometric measurements with cardiovascular risk factors.

### Non response

In total 6,161 mothers and children were approached to participate in phase 3 of the study. Of this population 4,687 children were from Dutch, Turkish, Moroccan, Black-African or Black-Caribbean origin. The ethnic groups included in this study (response group n=2,397 (51% of 4,687)) were comparable to the ethnic groups excluded from the study (n=2,290 (49% of 4,687)) with respect to almost all measured sociodemographic and birth outcome variables (all p values > 0.06). However, the Dutch and Turkish mothers in the response group were slightly older (Dutch: 32.8 vs. 31.5 years and Turkish: 27.3 vs. 25.6 years (p<0.04)) and Dutch mothers were higher educated (>10 years after primary school: Dutch: 64.4% vs. 50.5%; African descent (p<0.001)). Furthermore, the percentage Moroccan boys was higher in the response group (56.2% versus 48.4% (p=0.048))

### Data analysis

Ethnic differences in body size and BP were examined with χ^2^-tests (categorical data) or ANOVA (continuous data). Linear regression was used to examine the ethnic specific associations between the body size measures (BMI, WHtR and FMI) and SBP and DBP, adjusted for confounders (child’s age, sex height and birth weight). The proportion of variance explained (R2) was calculated to determine which body size measure best predicts BP. Since all associations were similar in girls and boys (all p >0.1 for interaction) no stratification was made for sex.

Restricted cubic splines (RCS) with 3 knots were used in the regression analysis to examine and characterise an association that is suspected to be non-linear. We used higher order piecewise polynomials (splines) to accommodate potential changes in the direction of the association across the exposure distribution. These techniques allow for graphical representation of the results across the exposure and outcome distributions. If the results for a given model indicated that a linear model provided an adequate fit (Wald *χ*^2^ p-value > 0.05), we reported the results from a linear model.

Analyses were stratified by ethnicity and a formal interaction test was used to establish p-values for the null hypothesis of no difference in association (linear or non-linear) between the ethnic groups (exposure ethnicity interaction).

Statistical analyses were conducted using *R* 2.13.1. A p-value <0.05 was regarded as statistically significant.

## Results

Table 1 presents the characteristics of the children at age 5-6 years, stratified for ethnicity. The Dutch children were younger, had a higher birth weight and generally had a leaner body size (BMI, WHtR and FMI) and lower SBP and DBP values (all p-values <0.001). The Black-African (119.3±6.1) and Black-Caribbean (119.1±5.9) were taller compared to the ethnic Dutch children (116.9±5.7). The percentage of pre-hypertension (according to reference guidelines [2]) was higher in the Moroccan (16.7%) and Turkish group (16.2%) compared to the Dutch group (11.1%) (χ^2^ p=0.04). In addition, the percentage of prehypertension was higher in overweight children compared to lean children (20.2% versus 11.3%; p<0.001).

**Table 1:** Characteristics of the study sample by ethnic group

	**Dutch n=1923**	**Turkish n=99**	**Moroccan n=187**	**Black-African n=67**	**Black-Caribbean n=121**	**sign**
	**Mean (SD) or %**					
Age (years)	5.7 (0.5)	5.9 (0.5)†	6.1 (0.6)$	5.9 (0.6)†	5.9 (0.5)†	***
Sex (% boys)	51.2	45.2	56.2	52.2	48.8	
Birth weight (gr)	3522 (539)	3395 (469)	3381 (557)	3398 (596)	3276 (604)	***
Height (cm)	116.9 (5.7)	116.7 (5.3)	118.1 (6.4)	119.3 (6.1)$	119.1 (5.9)†	***
Weight (kg)	21.1 (2.9)	22.5 (3.8)$	23.1 (4.3)†	23.1 (4.8)†	22.7 (4.1)†	***
BMI (kg/m^2^)	15.4 (1.3)	16.5 (2.0)$	16.5 (1.9)†	16.1 (2.2)†	15.9 (1.8)†	***
%Overweight/obese‡	6.6	27.3	26.2	20.9	16.5	***
WHtR	0.45 (0.03)	0.47 (0.04)†	0.46 (0.03)†	0.45 (0.04)	0.45 (0.03)	***
FMI (kg/m^2^)	3.1 (1.1)	4.0 (1.7)†	4.2 (1.7)†	3.8 (1.8)†	3.7 (1.7)†	***
Systolic BP (mmHg)	97 (9)	100 (9)	100 (9)$	99.0 (9.1)	99.1 (8.9)	***
Diastolic BP (mmHg)	57 (8)	60 (8)$	60 (8)†	60.5 (7.3)†	60.5 (7.5)†	***
Pre-hypertension % yes§	11.1	16.2	16.7	16.7	13.2	*

Abbreviations: *BMI* body mass index, *WHtR* waist-to-height-ratio, *FMI* fat mass index, *BP* blood pressure.

Significance level * p<0.05, ** p<0.01, *** p<0.001.

†Significantly different compared with ethnic Dutch children.

‡According to Cole et al.^22^.

§According to the Fourth Report on the diagnosis, evaluation, and treatment of high blood pressure in children and adolescents.^2^.

### Ethnic differences in the association between body size and BP

**Figure 2:**
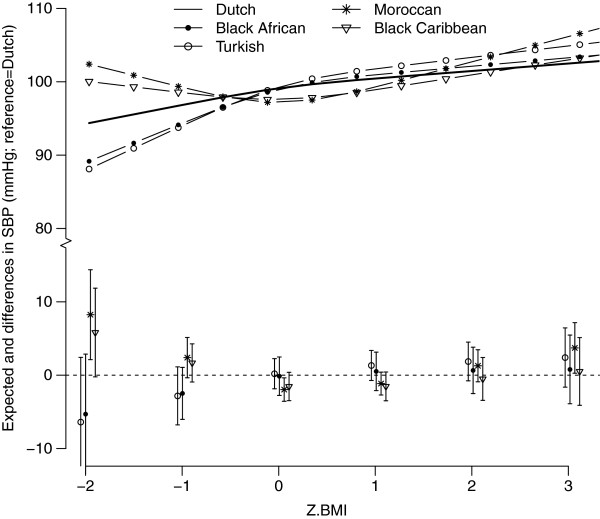
**The expected (lines) and mean differences (Dutch=reference) between z-BMI and SBP for the different ethnic groups, adjusted for sex, mean age (5.7 years), height (117 cm) and mean birth weight (3489 g).** The lines (top part) represent the expected SBP values for each ethnic group. The bottom part represents the mean differences in SBP for each ethnic group compared to the ethnic Dutch group (reference group) including the 95% confidence intervals. Both are plotted as a function of child’s BMI z-score.

**Figure 3:**
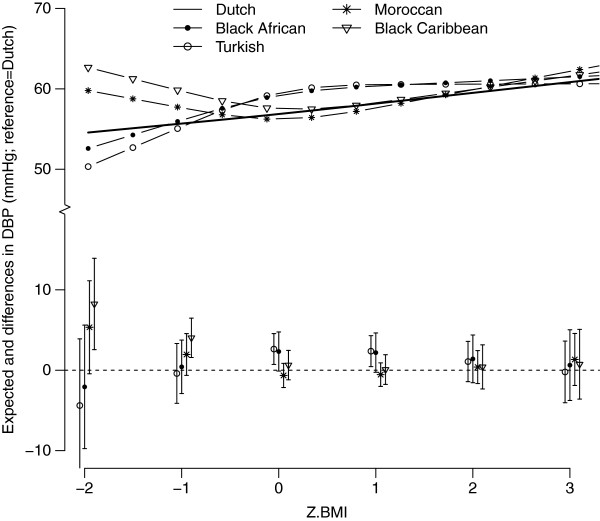
**The expected (lines) and mean differences (Dutch=reference) between z-BMI and DBP for the different ethnic groups, adjusted for sex, mean age (5.7 years), height (117 cm) and mean birth weight (3489 g).** The lines (top part) represent the expected DBP values for each ethnic group. The bottom part represents the mean differences in DBP for each ethnic group compared to the ethnic Dutch group (reference group) including the 95% confidence intervals. Both are plotted as a function of child’s BMI z-score.

The associations between BMI and BP differed between the ethnic groups (p for interaction SBP=0.001 and DBP=0.01); Figure 2 and 3 present the non-linear associations. Turkish children showed a steeper increase in SBP with increasing BMI compared to the Dutch children. SBP tended to be higher in the Black-Caribbean and Moroccan children with extreme low BMI values, and also for Moroccans with extremely high BMI values (Figure 2). At average BMI levels (z-BMI=0), the associations with SBP were highest for the Turkish (β: 2.46 mmHg; 95%CI: 1.20-3.72) and Moroccan children (β: 2.37 mmHg; 95%CI: 1.33-4.42) (Table 2). The pattern of DBP was similar to that of SBP (Figure 3): at average BMI levels (z-BMI=0), the effect sizes for DBP were highest for the Dutch children (β: 1.32 mmHg; 95%CI: 0.74-1.90) (Table 2).

**Table 2:** Association between BMI, WHtR and FMI with BP at age 5 years by ethnicity

	**Dutch**		**Turkish**		**Moroccan**		**Black-African**		**Black-Caribbean**	
	**Beta (95%CI)**	**R2†**	**Beta (95%CI)**	**R2**	**Beta (95%CI)**	**R2**	**Beta (95%CI)**	**R2**	**Beta (95%CI)**	**R2**
**BMI z-score**‡										
Systolic BP	1.31 (0.71, 1.92)	0.028	2.46 (1.20, 3.72)	0.131	2.37 (1.33, 4.42)	0.122	1.83 (0.36, 3.30)	0.086	1.48 (0.18, 2.28)	0.037
Diastolic BP	1.32 (0.74, 1.90)	0.016	1.09 (-0.22, 2.41)	0.047	1.19 (0.36, 2.02)	0.052	1.13 (-0.18, 2.44)	0.012	0.59 (-0.58, 1.77)	0.033
**WHtR z-score**										
Systolic BP	1.43 (0.91, 1.94)	0.024	1.24 (-0.33, 2.81)	0.022	1.93 (0.51, 3.36)	0.035	1.50 (-0.46, 3.46)	0.027	1.02 (-0.54, 2.58)	0.011
Diastolic BP	1.02 (0.53, 1.52)	0.008	0.56 (-1.03, 2.15)	0.009	1.40 (0.31, 2.50)	0.029	1.25 (-0.45, 2.94)	0.034	0.42 (-1.00, 1.84)	0.007
**FMI z-score**										
Systolic BP‡	0.84 (0.23-1.45)	0.006	2.41 (1.09-3.73)	0.125	1.99 (0.81-3.18)	0.098	1.98 (0.17-3.78)	0.057	0.89 (-0.42, 2.20)	0.018
Diastolic BP	1.10 (0.64-1.56)	0.010	1.21 (-0.19-2.62)	0.023	1.42 (0.49-2.34)	0.038	2.14 (0.58-3.70)	0.043	0.77 (-0.42, 1.95)	0.009

Abbreviations: *BMI* body mass index, *WHtR* waist-to-height ratio, *FMI* fat mass index, *BP* blood pressure.

Covariates include: child's age, sex, birth weight and height.

Explained variance (R2) for child’s age, sex, birth weight and height: Dutch SBP: 0.098 and DBP: 0.043, Turkish: SBP: 0.140 and DBP: 0.098, Moroccan SBP: 0.067 and DBP: 0.185, Black-African SBP: 0.255 and DBP: 0.146 and Black-Caribbean SBP: 0.225 and DBP: 0.113.

†R2=explained variance for BMI, WHtR or FMI.

‡Estimated Betas for z-BMI=0 or z-FMI=0.

Formal testing revealed no ethnic differences in the association of WHtR with SBP and DBP; in the stratified analysis the associations were significant only in Dutch children (SBP β: 1.43 mmHg; 95%CI: 0.91-1.94 and DBP β: 1.02 mmHg; 95%CI: 0.53-1.52) and Moroccan children (SBP β: 1.93 mmHg 95%CI: 0.51-3.36 and DBP β: 1.40 mmHg: 95%CI: 0.31-2.50) (Table 2).

**Figure 4:**
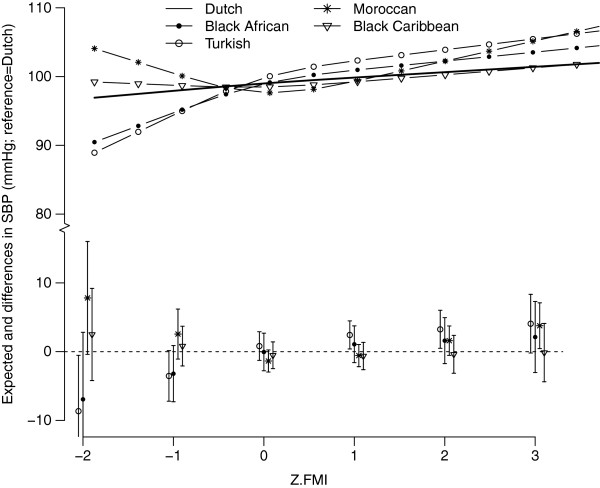
**The expected (lines) and mean differences (Dutch=reference) between z-FMI and SBP for the different ethnic groups, adjusted for sex, mean age (5.7 years), height (117 cm) and mean birth weight (3489 g).** The lines (top part) represent the expected SBP values for each ethnic group. The bottom part represents the mean differences in SBP for each ethnic group compared to the ethnic Dutch group (reference group) including the 95% confidence intervals. Both are plotted as a function of child’s FMI z-score.

The association between FMI and SBP differed between the ethnic groups (p for interaction= 0.01) with nonlinear association (Figure 4). Turkish children showed a steeper increase in SBP with increasing FMI compared to Dutch children. A convex curve was seen for the Moroccan children with higher SBP in the extremes of the FMI range. At average FMI levels (z-FMI=0), the associations with SBP were highest for Turkish children (β: 2.41 mmHg: 95%CI: 1.09-3.73) (Table 2).

In general, BMI, WHtR and FMI had a relatively weak association with BP in Black- Caribbean children; most associations were non-significant and tended towards the null.

Although the additional proportions of explained variances were low for all body size measures, the strongest associations and highest additional explained variances were found between BMI and BP (Table 2).

## Discussion

This cross-sectional study explored ethnic-specific associations between three measures of body size (BMI, WHtR and FMI) and BP (SBP and DBP) in children aged 5-6 years. In addition, we compared BMI with WHtR and FMI as determinants of BP. The data revealed ethnic differences in the association between body size and BP: (i) body size had a relatively large effect on BP in Turkish children, indicating higher BP (mainly SBP) levels with increasing BMI and FMI, (ii) Black- Caribbean and Moroccan children showed high BP at low BMI, whereas at low FMI this effect was seen only in Moroccan children. Moroccan children also showed higher SBP at high BMI and FMI, (iii) in general, the strongest associations with BP were found for BMI in all ethnic groups.

Studies on ethnic differences in the association between body size and BP are scarce, and the necessity to account for ethnic differences remains debatable [3,7,19-21]. For example, Rosner et al. reported an interaction with ethnicity in the association between BMI and BP in Black and White children in the USA [20]. Comparable results were found in a cohort with predominantly Caucasian and Asian ethnicities [3]. Both studies concluded that ethnicity may be an effect modifier in the association of body size with BP, confirmed by a study which reported higher risk for elevating SBP for South East Asian children compared to their Australian counterparts when they gain in body mass or WC [19]. On the other hand, Harding et al. found only minor ethnic-specific effects for BP in a multi-ethnic cohort of UK adolescents [7]. To our knowledge this is the first population study located in Europe to include Turkish and Moroccan children at young age.

Ethnic-specific associations were found for BMI and FMI with BP. Biological (differences in body composition [22-24]), behavioural and environmental factors, including diet and nutrition (especially salt intake [30]), may play a role in an explanation for these differences. Hypothetically, the convexity in the association between BP and body size for the Moroccan and Black-Caribbean children might have its origin in early life. We adjusted for birth weight, which is an independent risk factor for high BP. There is evidence that rapid postnatal growth is also associated with higher BP, even in the absence of obesity [31,32]. Both Black-Caribbean and Moroccan children have higher weight gain in the first months of life [33]. In addition, salt sensitivity might derive from under-nutrition *in utero*, reducing nefron number and increasing the risk for development of high BP [34]. We have to keep in mind that, although the betas were significant, the absolute numbers were low (both in the low and high BMI ranges), and we cannot rule out a spurious finding. These results need to be confirmed in additional studies before definite conclusions can be drawn.

Several mechanisms can play a role in the association between body size and elevated BP [35,36]. There is evidence that obesity and central fat mass impairs microvascular function that, in turn, may result in the development of hypertension [36,37]. Obesity leads to an enhanced secretion of adipokines and inflammatory cytokines, which interfere with the normal physiological process, leading to higher BP [36]. There is no evidence that these mechanisms work differently in different ethnic groups, but Ujcic-Voortman et al. reported higher C-reactive protein (CRP) levels, a marker of inflammation, in Turkish and Moroccan adults [38]. Whether these differences already appear in children is unknown, although the finding of Ujcic-Voortman et al. suggests that differences in CRP levels might play a role in the greater increase in BP by increasing body size in the Turkish children. Further research is needed to substantiate this hypothesis.

Earlier reports in adults and adolescents show that BP tended to be less influenced by BMI in Blacks compared to Whites [20,39]. We also found small regression coefficients in the Black-Caribbean children, although these were not significantly different from the Dutch. Black-Africans in the present study showed a highly similar association with the Dutch children. Even though we cannot rule out a lack of statistical power to detect this effect, differences in age range might explain the absence of an ethnic-specific effect between these groups in our study.

Both cross-sectional and longitudinal studies have investigated the association between BMI and WHtR (or WC) with BP, but rarely considered the use of more directly assessed fat mass (or FMI). Both Brion et al. and Lawlor et al. used FM determined by dual energy X-ray absorptiometry, to analyze the association between fat mass and BP and found positive associations [13,40]. Studies using BIA to determine FM or derivates of FM (e.g. FM%) also found correlations with BP [17,41]. To date, there is no consensus as to which body size measure is the strongest predictor for BP.

In the present study, BMI tended to be more strongly associated with BP in all ethnic groups compared to WHtR and FMI. Nevertheless, none of the body size measures showed superior discriminatory capability, and the predictive value of these measures was low. This suggests that, at this young age, other physiological factors may be more important determinants for BP. Due to the heterogeneous nature of the methodology and age ranges, it is difficult to compare magnitudes of associations with other studies. However, the calculated explained variances agree well with previous studies [42,43]. Changes in BMI over time, as seen in a longitudinal study of Mirzaei et al, might be a better predictor for BP [16]. However, this finding is not confirmed within data from the ALSPAC study finding similar associations with BP and changes in BMI, WC and fat mass in adolescence [13].

This large multi-ethnic cohort study allowed collecting extensive anthropometric and BP data, measured by trained research assistants following standardized protocols. We did not adjust for education level, as a measure for SES, and maternal BMI, because these factors are determinants of child’s body size [44,45] and are therefore in the causal pathway. In Western countries, such as the Netherlands, lower socio-economic status is strongly associated with higher BMI [44] and, in our opinion, adjusting for these factors would lead to overcorrection. However, when we included these factors in our model, we found comparable results (data not shown).

When interpreting our results, some limitations should be taken into account. First, due to small ethnic groups, we couldn’t analyse associations with BP separately for lean and overweight children. However, by using techniques that controlled for non-linearity we were able to visualize the non-linear association between BMI and FMI with SBP and/or DBP across the whole body size range. Second, WC (used to calculate WHtR) is difficult to measure. Especially in overweight children, variability in measurement may increase [46]. However, WC as measured in the present study represents the measurement sites most closely [47]. Third, measuring BP in children is more difficult than in adults, e.g., readings are likely to be falsely elevated in children who find it difficult to relax their arm during the measurement. To control for falsely high or low blood pressures, the BP measurements were performed according to a standardized protocol which included a test measurement (10 min in supine position and 5 min in sitting position) before the actual measurements were taken [48]. Finally, as far as we know, ethnic-specific equations for children aged 5-6 years to calculate fat mass with BIA are lacking. Despite that we used an equation validated for children aged 4-7 years [28], we cannot rule out possible under- or overestimation of FMI in the non-Caucasian groups.

In conclusion, we found evidence for ethnic-specific patterns in the associations between BMI and FMI and BP, with increased risk for elevated BP in the Turkish children in particular. Because, compared to the Dutch children, Turkish children have more overweight and higher blood pressure even at the same level of overweight; Turkish children may be more prone to develop hypertension. Longitudinal studies will show whether these differences at age 5-6 years track into adulthood. The higher BP in the Black-African and Moroccan children with low BMI needs further elucidation.

Among the various measures of body size, BMI appeared to be the most reliable determinant of BP in any ethnic group. This implies that in clinical practice and public health surveillance, at least in this age group, the use of more sophisticated measurements of fat mass and adiposity distribution are unlikely to be needed to screen for elevated BP. However, due to the low predictive ability of BMI to predict BP, BMI might not be the best selection method either to screen for elevated BP. Future studies should not only focus on different body size measurements, but also on other important determinants of BP such as, for example, diet.

## Competing interest

The authors declare that they have no competing interest.

## Authors’ contributions

MdH and TV developed the concept of the present study as part of the ABCD-study. MdH conducted the analyses and drafted the manuscript. All authors provided statistical advice, contributed to interpreting the results and writing the article. All authors read and approved the final manuscript.
